# Community-based surveillance of norovirus disease: a systematic review

**DOI:** 10.1186/s12879-017-2758-1

**Published:** 2017-09-29

**Authors:** Thomas Inns, John Harris, Roberto Vivancos, Miren Iturriza-Gomara, Sarah O’Brien

**Affiliations:** 1 Institute of Psychology, Health and Society, University of Liverpool, Liverpool, UK; 2 NIHR Health Protection Research Unit in Gastrointestinal Infections, University of Liverpool, Liverpool, UK; 3 Field Epidemiology Service, Public Health England, London, UK; 4 NIHR Health Protection Research Unit in Emerging and Zoonotic Infections, University of Liverpool, Liverpool, UK; 5 Institute of Infection and Global Health, University of Liverpool, Liverpool, UK

**Keywords:** Norovirus, Surveillance, Community, Gastroenteritis, Systematic review

## Abstract

**Background:**

Norovirus is a common cause of infectious gastrointestinal disease. Despite the increased ability to detect norovirus in affected people, the number of reported cases and outbreaks in the community is still substantially underestimated. We undertook a systematic review to determine the nature, scope and scale of community-based surveillance systems which capture information on norovirus disease.

**Methods:**

We searched MEDLINE, EMBASE and Scopus for studies published between 01 January 1995 and 31 December 2015, using terms relating to norovirus and surveillance. Publications were screened independently by two reviewers using exclusion criteria. Data extraction from included papers was performed using a standardized data extraction form. Outcomes were descriptions of the methods reported in included papers, and any estimates of incidence rate of norovirus disease in each community, stratified by age.

**Results:**

After exclusions, we reviewed 45 papers of which 23 described surveillance studies and 19 included estimates of incidence. The estimates of incidence varied by outcome measure, type of laboratory test and study population. There were two estimates of norovirus hospitalisation; 0.72 and 1.04 per 1000 person-years. Estimates of norovirus disease ranged between 0.024 cases per 1000 person-years and 60 cases per 1000 person-years and estimates of all gastroenteritis varied between 49 and 1100 cases per 1000 person-years.

**Conclusions:**

Our systematic review found few papers describing community-based surveillance for norovirus disease. Standardised age-specific estimates of norovirus incidence would be valuable for calculating the true global burden of norovirus disease; robust community surveillance systems would be able to produce this information.

**Trial Registration:**

PROSPERO 2016:CRD42016048659.

**Electronic supplementary material:**

The online version of this article (10.1186/s12879-017-2758-1) contains supplementary material, which is available to authorized users.

## Background

Norovirus infection is the most common cause of infectious gastrointestinal disease in the United Kingdom (UK) and many other countries [1, 2]. Globally, it is estimated to be associated with 18% of all cases of acute gastroenteritis [3]. Norovirus is a common cause of gastroenteritis outbreaks in healthcare settings [4]. Outbreaks are difficult to control in enclosed settings and the evidence for the effectiveness of infection control methods remains inconclusive [5, 6]. The infection is typically mild and self-limiting and people who are infected rarely have contact with medical services; further detail on the clinical manifestations of norovirus has been described elsewhere [1]. Some groups, particularly the elderly, can have longer episodes of illness [7], and are at risk of more serious outcomes [8].

Surveillance of norovirus disease is essential for providing information for norovirus prevention and control [9]. Different types of surveillance system are used and have been described elsewhere [5]. Norovirus surveillance is largely based on laboratory diagnosis and the ability to detect norovirus in affected people has increased with the adoption of more sensitive molecular methods [10]. There is evidence that the number of reported cases and outbreaks in the community is substantially underestimated; and that this underestimation is greater in the community than hospital settings [5, 11, 12], but there is little evidence of the type and variety of community-based norovirus surveillance systems. We were prompted to undertake this research to address this gap.

The aim of this research is to determine the nature, scope and scale of community-based surveillance systems which capture information on norovirus disease. To do this, we undertook a systematic review according to the criteria of the Preferred Reporting Items for Systematic Reviews and Meta-Analyses (PRISMA) statement [13]. In order to determine the nature, scope and scale of community-based surveillance of norovirus disease, we described the methods reported in papers included in the review, as a primary objective. A secondary objective was to capture the incidence rate of norovirus disease in each setting.

## Methods

### Protocol and registration

The review was registered on the PROSPERO International prospective register of systematic reviews on 03 October 2016 (PROSPERO 2016:CRD42016048659) [14]. The review protocol followed the PRISMA checklist (Additional file 1).

### Eligibility criteria

Studies published between 01 January 1995 and 31 December 2015. No explicit geographical restrictions were applied. Only studies published in English were eligible. We excluded the following types of publication: studies of illness in persons residing in primary care settings, reports or reviews of outbreak investigations, review papers, editorials, conference abstracts or proceedings, randomized clinical trials or case reports, environmental surveillance, economic analyses, studies based on asymptomatic infections, surveys of molecular epidemiology or seroprevalence surveys.

### Information sources

We searched the following electronic databases: MEDLINE, EMBASE and Scopus. The last date searched was 16 August 2016.

### Search

We used the following search terms: (norovirus.ab,ti. OR (norwalk-like adj1 virus).ab,ti. OR (norwalk-like adj1 disease*).ab,ti. OR norwalk.ab,ti. OR small round structured virus.ab,ti. OR winter vomiting disease.ab,ti.) AND surveillance.ab,ti. The search terms were piloted prior to selection and are comprised of specific norovirus terms. The search terms for MEDLINE were developed initially. Terms were combined using Boolean operators. The same terms were used to search Scopus. When the searches were run in MEDLINE, each term was searched for within the title and abstract of the documents contained in each database; in Scopus, terms were searched within the title, abstract and keywords.

### Study selection

All references identified by the search strategy were imported into the reference management programme EndNote X7 (Clarivate Analytics, USA). Using this software, publications from the different databases were combined and deduplicated. These publications were then screened applying the exclusion criteria. This screening was conducted independently by two reviewers (TI and JPH) to ensure the criteria were applied consistently. Differences between reviewers on first screening were reconciled by discussion between the two reviewers. Full texts of all studies meeting the title and abstract screening criteria were examined independently by the two reviewers (TI and JPH) using a standardised eligibility form. Final agreement on study inclusion was determined through consensus between the two reviewers (TI and JPH).

In order to maximise the proportion of eligible studies included in the review, the reference lists of studies that met the inclusion criteria were searched to identify potentially relevant articles not included be the database searches. When potentially relevant articles were identified in this way, the two reviewers (TI and JPH) searched for abstracts and then screened in the same fashion as those identified by the database search. The full text of any abstract that met the eligibility criteria was assessed using the standardised eligibility form; final agreement was determined through consensus between the two reviewers.

### Data collection process and data items

Data extraction from included papers was performed using a standardized data extraction form. The following data were extracted (where available): year published, predominant study type, surveillance type (active or passive), study setting, time period, study duration, geography (country, region), case definition, laboratory testing methods, proportion of norovirus detections, use of further typing methods, population age range, study population, person-time of study population, number of cases, incidence rate with 95% Confidence Interval. We defined the surveillance type as active if the person-time at risk was actively enumerated.

### Summary measures

Outcomes were descriptions of the methods reported in included papers, and any estimates of incidence rate of norovirus disease in each community, stratified by age. Estimates of incidence rates were not pooled between studies.

### Synthesis of results

We compared methods used in included papers. We described the study design and methods used, the population under surveillance, the date of publication, the location of study and compared incidence rates in various studies, stratified by age where available.

## Results

### Study selection

**Fig. 1:**
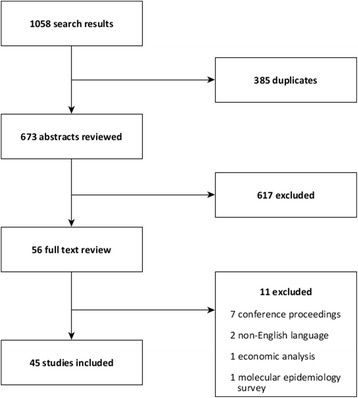
Study selection, systematic review of community-based surveillance of norovirus disease (*n* = 1058)

The first searches identified 1058 papers; following de-duplication this was reduced to 673 publications. After review of the title and abstract, 56 publications were included and 617 excluded. Of the 56 publications subject to full text review, 11 were excluded and 45 included in this systematic review (Fig. 1). Of the 11 papers excluded after full text review, seven were conference proceedings, two were non-English language, one was an economic analysis and one was a survey of molecular epidemiology.

### Characteristics of included studies

**Fig. 2:**
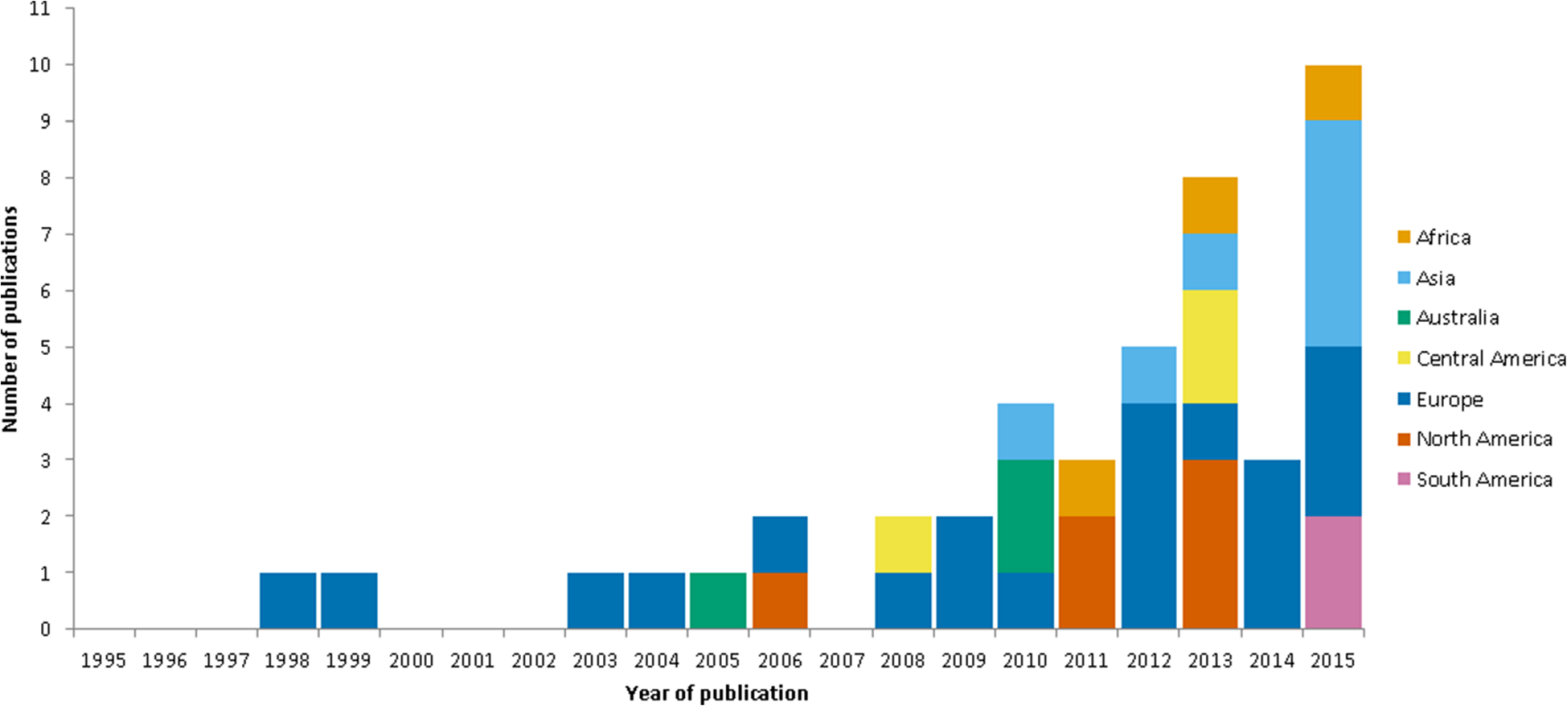
Reviewed studies; shown by year of publication and geography of study location (*n* = 44*). *One study published in 2015 was based in several sites across the world

The number of publications regarding the community-based surveillance of norovirus disease increased over the 20 year period included in this review; two thirds of papers (*n* = 30) were published since 2010. Papers based in community settings in Europe (*n* = 20) were most frequent, the majority of these being from the United Kingdom (*n* = 7) and France (*n* = 4). Other countries with multiple published studies include the United States of America (*n* = 6), Australia (*n* = 3) and China (*n* = 3). Figure 2 shows the distribution of publications over time and by geography of study location.

A total of 23 publications described surveillance studies; 18 of these described surveillance of individuals, five described surveillance of norovirus outbreaks in the community. Other community-based publications included cohort studies (*n* = 14), cross-sectional surveys (*n* = 7) and one case–control study in a community setting. A breakdown of included publications by study type is shown in Table 1.

**Table 1:** Reviewed publications, by study type and outcome (*n* = 45)

First Author	Year Published	Study Type	Surveillance Type	Incidence outcome measure	Study country	Study Duration (Years)
Dedman [15]	1998	Surveillance	Passive	Norovirus disease	UK	5
Wheeler [47]	1999	Cohort	Active	All gastroenteritis; Norovirus disease	UK	3
Lopman [16]	2003	Surveillance	Passive	–	UK	7
Froggatt [17]	2004	Surveillance	Passive	–	UK	0.5
Sinclair [48]	2005	Cohort	Active	–	Australia	1.5
Medici [42]	2006	Cohort	Passive	–	Italy	1
Vernacchio [38]	2006	Cohort	Active	All gastroenteritis	USA	1.5
Bucardo [43]	2008	Cohort	Passive	–	Nicaragua	1
Gomara [39]	2008	Cohort	Active	–	UK	1.25
Huhulescu [49]	2009	Cohort	Active	All gastroenteritis	Austria	1
Iturriza-Gomara [40]	2009	Cohort	Active	All gastroenteritis	UK	1.66
Gauci [52]	2010	Cross-sectional survey	Active	All gastroenteritis	Malta	1.66
Kirk [27]	2010	Outbreak surveillance	Passive	–	Australia	6
Kirk [50]	2010	Cohort	Active	–	Australia	1
Liu [33]	2010	Surveillance	Passive	–	China	1
Moyo [57]	2011	Cross-sectional survey	Passive	–	Tanzania	0.25
Scallan [19]	2011	Surveillance	Passive	–	USA	8
Vega [22]	2011	Outbreak surveillance	Passive	–	USA	1
Baumann-Popczyk [53]	2012	Cross-sectional survey	Active	All gastroenteritis	Poland	1
Oldak [35]	2012	Surveillance	Passive	All gastroenteritis	Poland	1
Ouyang [34]	2012	Surveillance	Passive	–	China	1
Tam [11]	2012	Cohort	Active	All gastroenteritis; Norovirus disease	UK	1.33
Thouillot [24]	2012	Outbreak surveillance	Passive	–	France	0.33
Ahmed [54]	2013	Cross-sectional survey	Active	All gastroenteritis	Dominica	1.5
Gould [23]	2013	Outbreak surveillance	Passive	–	USA	10
Ingram [55]	2013	Cross-sectional survey	Active	All gastroenteritis	Barbados	1
Nahar [32]	2013	Surveillance	Passive	–	Bangladesh	1
Payne [31]	2013	Surveillance	Active	Norovirus hospitalisation	USA	2
Saupe [21]	2013	Surveillance	Passive	–	USA	1.25
Trainor [36]	2013	Surveillance	Passive	–	Malawi	10
Verhoef [56]	2013	Cross-sectional survey	Passive	Norovirus disease	Netherlands	1
Arena [20]	2014	Surveillance	Passive	All gastroenteritis	France	2
Barret [25]	2014	Outbreak surveillance	Passive	–	France	1.5
Bernard [18]	2014	Surveillance	Passive	Norovirus disease	Germany	8
Anders [45]	2015	Cohort	Active	All gastroenteritis	Vietnam	4
Ballard [51]	2015	Cohort	Active	All gastroenteritis; Norovirus disease	Peru	9
Enserink [41]	2015	Cohort	Active	All gastroenteritis	Netherlands	3
Fernandez [37]	2015	Case–control	Passive	–	Colombia	not stated
Gaspard [26]	2015	Outbreak surveillance	Active	–	France	6
Lekana-Douki [44]	2015	Cohort	Passive	–	Gabon	1.25
Leshem [30]	2015	Surveillance	Active	Norovirus hospitalisation	Isreal	7
Platts-Mills [46]	2015	Cohort	Active	–	multi-site	3
Sakon [28]	2015	Surveillance	Passive	–	Japan	10
Thongprachum [29]	2015	Surveillance	Passive	–	Japan	4
Xue [58]	2015	Cross-sectional survey	Active	–	China	2

Description of methods reported in community-based norovirus surveillance.

Reports of community-based surveillance of laboratory reports of norovirus infection were published from England and Wales [15–17], Germany [18] and the United States of America (USA) [19]. Surveillance using sentinel general practitioners (family doctors) was reported from France [20]. Surveillance of norovirus using a foodborne illness complaint system in the USA state of Minnesota [21]. Reports of the surveillance of norovirus outbreaks in the community were published from the USA [22, 23]. Norovirus outbreak surveillance in care homes was reported from France [24–26] and Australia [27].

A number of papers described the surveillance of cases of norovirus acquired in the community, through the surveillance of cases admitted to hospital from the community. Sentinel networks or small groups of hospitals published their findings from Japan [28, 29], Israel [30], USA [31] and Bangladesh [32]. Four publications reported surveillance at individual hospitals in China [33, 34], Poland [35] and Malawi [36]. Children under five years old were included in one case–control study in Colombia [37]. A total of 20 papers were classed as active surveillance and 25 were classed as passive surveillance.

Cohort studies captured surveillance data on norovirus disease in children in general practice [38–40], in day care [41] and in hospital [42–44]. Two birth cohort studies, one in Vietnam [45] and one across eight sites in South America, Africa and Asia [46]. Four cohort studies captured surveillance data on norovirus disease in all of those attending general practice [11, 47–49]; one cohort study was based in 16 care homes [50] and one a cohort study of military personnel [51]. A number of cross-sectional studies from different countries collected information on the general population [52–56]. One study was of hospitalised children [57] and another included hospital outpatients [58].

Of the 39 papers which covered the surveillance of individual cases, all but one used a laboratory test to confirm the presence of norovirus. Polymerase chain reaction (PCR) was used either alone or in combination with other methods by 29 of the reports; enzyme immunoassay (EIA) was used as the only method in two studies and electron microscopy (EM) as the only method in one study. The laboratory methods were either absent or unclearly defined in five studies. The case definition was based on the WHO definition of diarrhoea in 22 of the 39 studies; another symptomatic definition was used in 13 of the studies, one study used a virological case definition and the case definition was unclear in the remaining three studies. Regarding the surveillance of norovirus outbreaks, three of the six used the Centres for Disease Control and Prevention (CDC) outbreak definition [22, 23, 27]. Comparable definitions were used by the other three studies [24–26]. All norovirus outbreak surveillance systems used some form of laboratory confirmation; PCR was used alone or in combination for five studies, the lab method wasn’t specified in the other study.

### Estimates of the incidence rate of norovirus disease in each community

**Fig. 3:**
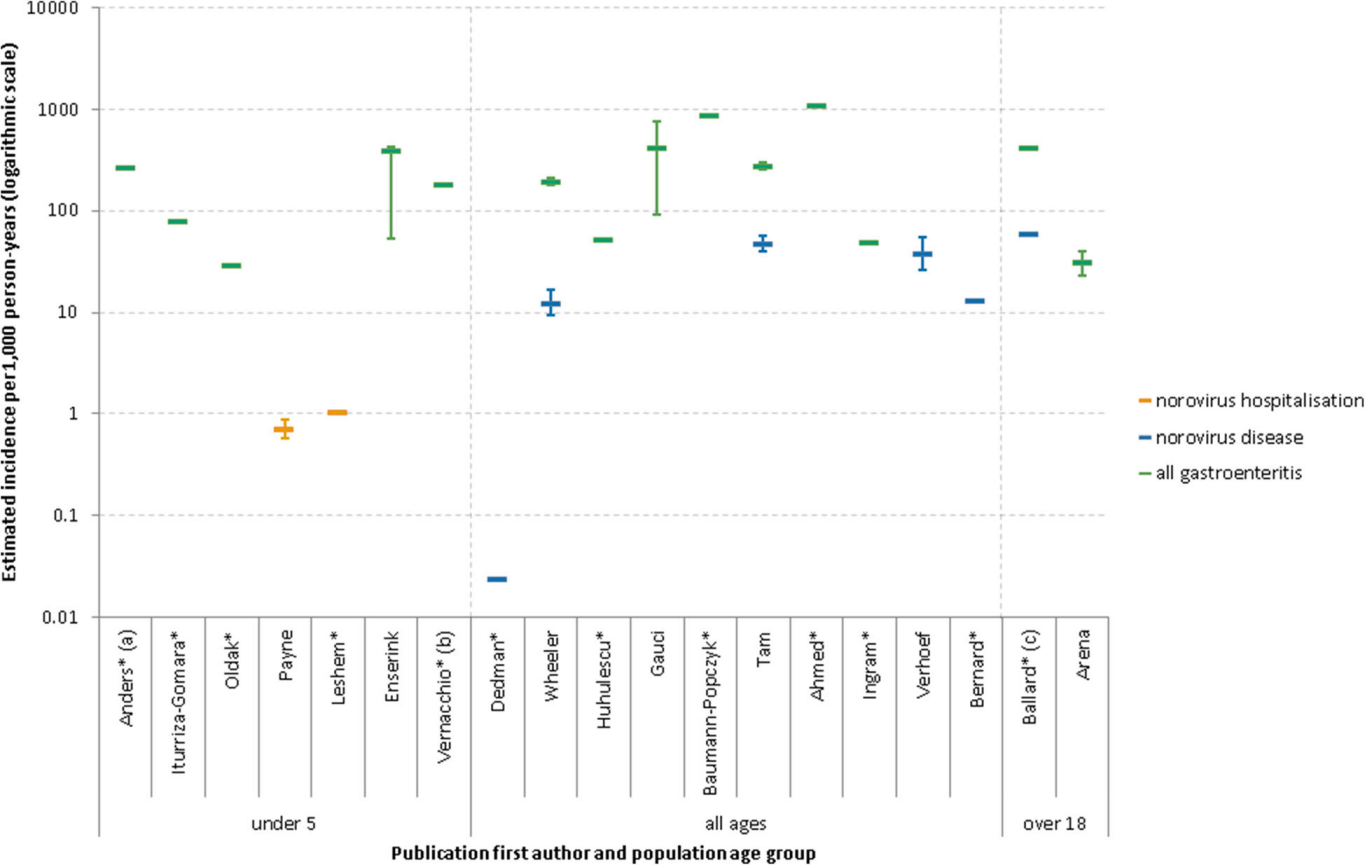
Studies with estimates of community incidence, shown by measured outcome and population age group (*n* = 19). *No published Confidence Interval estimate; (a) population under 1; (b) 6 months to 3 years old; (c) males aged 18 to 34

Publications included in this review measured outcomes that can be classified into three groups; norovirus hospitalisation, norovirus disease and all gastroenteritis. A total of 19 papers included estimates of incidence, of which seven also published Confidence Intervals. Figure 3 depicts the estimated incidence rates by outcome and study age group. Of the 19 papers, 14 were classed as active surveillance and clearly enumerated the person-time at risk.

Two papers used norovirus hospitalisation as an outcome, both were based in those aged under five and reported similar estimates; 0.72 [31] and 1.04 [30] hospitalisations per 1000 person-years. Six papers provided estimates of norovirus disease incidence. Estimates of norovirus disease ranged between 0.024 cases per 1000 person-years and 60 cases per 1000 person-years [11, 15, 18, 47, 51, 56].

Incidence rates of all gastroenteritis were estimated by 14 papers. Estimates in children under five ranged between 29.5 and 389 cases per 1000 person-years [35, 38, 40, 41, 45]. Estimates in all ages ranged between 49 and 1100 cases per 1000 person-years [11, 47, 49, 52–55]. One paper estimated the incidence rate of all gastroenteritis in long-term care facility (LTCF) resident as 0.64 cases per 1000 bed-days [50]. Another estimated the incidence rate of gastroenteritis outbreaks in LTCFs as 16.8 per 100 LTCFs per year [27].

## Discussion

Our systematic review has found few papers describing community-based surveillance for norovirus disease. We found surveillance based on individual laboratory reports were reported from four countries; England and Wales, Germany, France and the USA. Surveillance of outbreaks in care homes was reported from France and Australia. We found a number of hospital-based surveillance reports capturing illness acquired in the community; these tended to be based in a single or small number of hospitals and many were cross-sectional or cohort studies in a fixed time period. Several papers from the USA reported on the surveillance of outbreaks associated with food.

The small number of national surveillance systems reporting norovirus disease is likely to be related to the knowledge that most people do not access health care for a diagnosis [11]. One explanation could be the lack of statutory basis for norovirus reporting. The European Surveillance System (TESSy) is a system used by 7European Union (EU) Member States and European Economic Area (EEA) countries for the collection, analysis and dissemination of data on communicable diseases. Norovirus disease is not one of the 52 communicable diseases covered by this surveillance system [59]. Another reason could be the low priority of testing for norovirus with limited healthcare or laboratory resources. Due to the usually mild self-limiting nature of the disease, testing for norovirus may not be prioritised by healthcare providers. This is an issue in high-resource countries, but is particularly relevant in low-resource settings. This under-representation of developing countries may be partially addressed by plans for the inclusion of norovirus in the World Health Organisation (WHO) global rotavirus surveillance network [60], which includes a number of developing countries. The change in the number of papers published, whereby we observed that the majority have been published since 2010, is likely to be related to the change in testing from electron microscopy to more rapid and more sensitive techniques such as real-time polymerase chain reaction (RT-PCR) and EIA [61].

We found that estimates of the incidence rate of community-based norovirus disease had three outcome measures; norovirus hospitalisation, norovirus disease and all gastroenteritis. The two estimates of norovirus hospitalisation in children were both similar, around one case per 1000 person-years. Of the estimates of norovirus disease, one estimate was far lower (0.024 cases per 1000 person-years) than the others [15]. This much lower estimate is possibly due to the different diagnostic methods used; this is the earliest published study included in the review and at that time diagnosis of norovirus was by electron microscopy which is far less sensitive for detecting norovirus. This may also reflect changes in the criteria for testing for norovirus; as cheaper PCR tests have become more widespread, this may have led laboratories to widen the criteria for testing stool specimens for norovirus. The wide range of other estimates for norovirus disease (12.5–60 cases per 1000 person-years) and all gastroenteritis (29.5–1100 cases per 1000 person-years) probably reflects the different populations and age groups included. Unfortunately 12 of the publications included a point estimate, but did not include any estimate interval. Therefore, caution must be used when drawing conclusions from the differing estimates.

Subsequent to the period of this literature search, a report has been published on an enhanced surveillance system for norovirus in an area of China [62]. The rate of norovirus-associated diarrhoea that they observed was 89 cases per 1000 person-years (95% CI 82–97); this is higher than any of the estimates captured in this review, the next highest being 60 cases per 1000 person-years from a study in South America [51]. This higher rate in China may represent an increased incidence in this population, or could be a product of the extrapolations used to produce the estimate from the surveillance data.

In this review we were only able to include papers written in English due to resource limitations. As a consequence of not including those publications not in English, it is likely that we have under-represented the findings of countries where English is not widely spoken. In addition, some reports or descriptions of surveillance systems may be published on institute websites rather than indexed journals. This type of “grey literature” is difficult to search and capture in a systematic way, so is therefore excluded from this review, possibly affecting the representativeness of our findings. We were not able to undertake a meta-analysis of the norovirus incidence rates due to the extensive heterogeneity in study designs, laboratory methods, outcome measures and study populations. A meta-analysis to estimate the prevalence of norovirus in persons with acute gastroenteritis has previously been conducted [3].

Of the 45 publications included in this review, incidence estimates were only available for 19. Excluding those publications reporting on the surveillance of outbreaks, 20 publications did not include an estimate of incidence. Of the 19 papers reporting incidence estimates, 14 were classed as active surveillance as they clearly enumerated the person-time at risk in the population. The five papers classed as passive surveillance used population denominators which assume that all persons would have been captured in the surveillance system had they become a case. It has been shown that a large proportion of norovirus cases are not captured in national surveillance systems [11], so estimates from these passive systems have to be interpreted in this light. A number of the surveillance publications, particularly those based in hospitals, did not report or estimate a denominator population. Without a population denominator, it is not possible to calculate incidence rates. We would recommend that future publications of this kind include an estimate of the population denominator as good epidemiological practice, and to facilitate further research of this kind.

## Conclusions

In this systematic review, we found that despite norovirus being an important cause of acute gastroenteritis, in terms of number of cases that occur, few papers describe community-based surveillance for it, and a small number report any measure of norovirus incidence. Standardised age-specific estimates of norovirus incidence would be valuable for calculating the true global burden of norovirus disease; robust community surveillance systems would be able to produce this information.

## Additional file

Additional file 1: Completed PRISMA checklist. (DOC 62 kb)

## Abbreviations

CDC: Centres for Disease Control and Prevention
EEA: European Economic Area
EIA: Enzyme immunoassay
EM: Electron microscopy
EU: European Union
LTCF: Long-term care facility
PCR: Polymerase chain reaction
PRISMA: Preferred Reporting Items for Systematic Reviews and Meta-Analyses
RT-PCR: Real-time polymerase chain reaction
TESSy: The European Surveillance System
WHO: World Health Organisation

## References

[1] Atmar RL, Estes MK (2006). The Epidemiologic and Clinical Importance of Norovirus Infection. Gastroenterol Clin N Am.

[2] Patel MM, Hall AJ, Vinjé J, Parashar UD (2009). Noroviruses: A comprehensive review. J Clin Virol.

[3] Ahmed SM, Hall AJ, Robinson AE, Verhoef L, Premkumar P, Parashar UD, Koopmans M, Lopman BA (2014). Global prevalence of norovirus in cases of gastroenteritis: a systematic review and meta-analysis. Lancet Infect Dis.

[4] Meakins SM, Adak GK, Lopman BA, O'Brien SJ (2003). General outbreaks of infectious intestinal disease (IID) in hospitals, England and Wales, 1992–2000. J Hosp Infect.

[5] Harris JP (2016). Norovirus Surveillance: An Epidemiological Perspective. J Infect Dis.

[6] Harris JP, Lopman BA, O'Brien SJ (2010). Infection control measures for norovirus: a systematic review of outbreaks in semi-enclosed settings. J Hosp Infect.

[7] Lopman BA, Andrews N, Sarangi J, Vipond IB, Brown DWG, Reacher MH (2005). Institutional risk factors for outbreaks of nosocomial gastroenteritis: Survival analysis of a cohort of hospital units in South-west England, 2002–2003. J Hosp Infect.

[8] Harris JP, Edmunds WJ, Pebody RG, Brown DW, Lopman B (2008). Deaths from Norovirus among the Elderly, England and Wales. Emerging Infectious Disease journal.

[9] M'ikanatha NM, Lynfield R, Van Beneden CA, de Valk H: Infectious Disease Surveillance, 2nd edn. Chichester: Wiley-Blackwell; 2013.

[10] Rabenau HF, Stürmer M, Buxbaum S, Walczok A, Preiser W, Doerr HW (2003). Laboratory Diagnosis of Norovirus: Which Method Is the Best?. Intervirology.

[11] Tam CC, Rodrigues LC, Viviani L, Dodds JP, Evans MR, Hunter PR, Gray JJ, Letley LH, Rait G, Tompkins DS (2012). Longitudinal study of infectious intestinal disease in the UK (IID2 study): Incidence in the community and presenting to general practice. Gut.

[12] Harris JP, Adams NL, Lopman BA, Allen DJ, Adak GK (2014). The development of web-based surveillance provides new insights into the burden of norovirus outbreaks in hospitals in England. Epidemiol Infect.

[13] Liberati A, Altman DG, Tetzlaff J, Mulrow C, Gøtzsche PC, Ioannidis JPA, Clarke M, Devereaux PJ, Kleijnen J, Moher D. The PRISMA statement for reporting systematic reviews and meta-analyses of studies that evaluate healthcare interventions: explanation and elaboration. BMJ. 2009;339 10.1136/bmj.b2700 PMC2714672 19622552

[14] Inns T, Harris J, Vivancos R, Ituriza-Gomara M, O'Brien SJ: Community-based surveillance of norovirus disease: a systematic review. PROSPERO 2016:CRD42016048659. http://www.crd.york.ac.uk/PROSPERO/display_record.asp?ID=CRD42016048659. 10.1186/s12879-017-2758-1 PMC5622532 28962598

[15] Dedman D, Laurichesse H, Caul EO, Wall PG (1998). Surveillance of small round structured virus (SRSV) infection in England and Wales, 1990–5. Epidemiol Infect.

[16] Lopman BA, Adak GK, Reacher MH, Brown DWG (2003). Two epidemiologic patterns of Norovirus outbreaks: Surveillance in England and Wales, 1992–2000. Emerg Infect Dis.

[17] Froggatt PC, Vipond IB, Ashley CR, Lambden PR, Clarke IN, Caul EO (2004). Surveillance of Norovirus Infection in a Study of Sporadic Childhood Gastroenteritis in South West England and South Wales, during One Winter Season (1999–2000). J Med Virol.

[18] Bernard H, Höhne M, Niendorf S, Altmann D, Stark K (2014). Epidemiology of norovirus gastroenteritis in Germany 2001–2009: eight seasons of routine surveillance. Epidemiol Infect.

[19] Scallan E, Hoekstra RM, Angulo FJ, Tauxe RV, Widdowson MA, Roy SL, Jones JL, Griffin PM (2011). Foodborne illness acquired in the United States-Major pathogens. Emerg Infect Dis.

[20] Arena C, Amoros JP, Vaillant V, Ambert-Balay K, Chikhi-Brachet R, Jourdan-Da Silva N, Varesi L, Arrighi J, Souty C, Blanchon T, et al. Acute diarrhea in adults consulting a general practitioner in France during winter: Incidence, clinical characteristics, management and risk factors. BMC Infect Dis. 2014;574 10.1186/s12879-014-0574-4 PMC4220050 25358721

[21] Saupe AA, Kaehler D, Cebelinski EA, Nefzger B, Hall AJ, Smith KE (2013). Norovirus surveillance among callers to foodborne illness complaint hotline, Minnesota, USA, 2011–2013. Emerg Infect Dis.

[22] Vega E, Barclay L, Gregoricus N, Williams K, Lee D, Vinje J (2011). Novel surveillance network for norovirus gastroenteritis outbreaks, United States. Emerg Infect Dis.

[23] Gould LH, Walsh KA, Vieira AR, Herman K, Williams IT, Hall AJ, Cole D. Surveillance for foodborne disease outbreaks - United States, 1998–2008. MMWR Surveill Summ. 2013:62, 1. 23804024

[24] Thouillot F, Delhostal C, Edel C, Bettinger A, Pothier P, Ambert-Balay K, Meffre C, Alsibai S. Gastroenteritis outbreaks in elderly homes in the East of France during winter 2009/10: Aetiology research for a series of 37 outbreaks. Eur Secur. 2012, 17:9. 22401565

[25] Barret AS, Jourdan-da Silva N, Ambert-Balay K, Delmas G, Bone A, Thiolet JM, Vaillant V (2014). Surveillance for outbreaks of gastroenteritis in elderly long-term care facilities in France, November 2010 to May 2012. Euro Surveill.

[26] Gaspard P, Ambert-Balay K, Mosnier A, Aho-Glélé S, Roth C, Larocca S, Simon L, Talon D, Rabaud C, Pothier P (2015). Burden of gastroenteritis outbreaks: Specific epidemiology in a cohort of institutions caring for dependent people. J Hosp Infect.

[27] Kirk MD, Fullerton KE, Hall GV, Gregory J, Stafford R, Veitch MG, Becker N (2010). Surveillance for outbreaks of gastroenteritis in long-term care facilities, Australia, 2002–2008. Clin Infect Dis.

[28] Sakon N, Yamazaki K, Nakata K, Kanbayashi D, Yoda T, Mantani M, Kase T, Takahashi K, Komano J (2015). Impact of genotype-specific herd immunity on the circulatory dynamism of norovirus: A 10-year longitudinal study of viral acute gastroenteritis. J Infect Dis.

[29] Thongprachum A, Takanashi S, Kalesaran AFC, Okitsu S, Mizuguchi M, Hayakawa S, Ushijima H (2015). Four-year study of viruses that cause diarrhea in Japanese pediatric outpatients. J Med Virol.

[30] Leshem E, Givon-Lavi N, Vinjé J, Gregoricus N, Parashar U, Dagan R (2015). Differences in Norovirus-Associated Hospital Visits Between Jewish and Bedouin Children in Southern Israel. Pediatr Infect Dis J.

[31] Payne DC, Vinjé J, Szilagyi PG, Edwards KM, Staat MA, Weinberg GA, Hall CB, Chappell J, Bernstein DI, Curns AT (2013). Norovirus and medically attended gastroenteritis in U.S. children. N Engl J Med.

[32] Nahar S, Afrad MH, Begum N, Al-Mamun F, Sarker AK, Das SK, Faruque ASG, Pourkarim MR, Choudhuri MSK, Azim T (2013). High prevalence of noroviruses among hospitalized diarrheal patients in Bangladesh, 2011. J Infect Dev Countries.

[33] Liu LJ, Liu W, Liu YX, Xiao HJ, Jia N, Liu G, Tong YG, Cao WC (2010). Identification of Norovirus as the Top Enteric Viruses Detected in Adult Cases with Acute Gastroenteritis. Am J Trop Med Hyg.

[34] Ouyang Y, Ma H, Jin M, Wang X, Wang J, Xu L, Lin S, Shen Z, Chen Z, Qiu Z (2012). Etiology and epidemiology of viral diarrhea in children under the age of five hospitalized in Tianjin, China. Arch Virol.

[35] Oldak E, Sulik A, Rozkiewicz D, Liwoch-Nienartowicz N (2012). Norovirus infections in children under 5 years of age hospitalized due to the acute viral gastroenteritis in northeastern Poland. Eur J Clin Microbiol Infect Dis.

[36] Trainor E, Lopman B, Iturriza-Gomara M, Dove W, Ngwira B, Nakagomi O, Nakagomi T, Parashar U, Cunliffe N (2013). Detection and molecular characterisation of noroviruses in hospitalised children in Malawi, 1997–2007. J Med Virol.

[37] Fernández KP, Ulloa JC, Meneses M, Matiz LF, Gutiérrez MF (2015). Norovirus, the principal cause of viral diarrhea in two regions of Colombia. Univ Sci.

[38] Vernacchio L, Vezina RM, Mitchell AA, Lesko SM, Plaut AG, Acheson DWK (2006). Characteristics of persistent diarrhea in a community-based cohort of young US children. J Pediatr Gastroenterol Nutr.

[39] Gomara MI, Simpson R, Perault AM, Redpath C, Lorgelly P, Joshi D, Mugford M, Hughes CA, Dalrymple J, Desselberger U (2008). Structured surveillance of infantile gastroenteritis in East Anglia, UK: Incidence of infection with common viral gastroenteric pathogens. Epidemiol Infect.

[40] Iturriza-Gomara M, Elliot AJ, Dockery C, Fleming DM, Gray JJ (2009). Structured surveillance of infectious intestinal disease in pre-school children in the community: "The Nappy Study". Epidemiol Infect.

[41] Enserink R, Mughini-Gras L, Duizer E, Kortbeek T, Van Pelt W (2015). Risk factors for gastroenteritis in child day care. Epidemiol Infect.

[42] Medici MC, Martinelli M, Abelli LA, Ruggeri FM, Di Bartolo I, Arcangeletti MC, Pinardi F, De Conto F, Izzi G, Bernasconi S (2006). Molecular epidemiology of norovirus infections in sporadic cases of viral gastroenteritis among children in Northern Italy. J Med Virol.

[43] Bucardo F, Nordgren J, Carlsson B, Paniagua M, Lindgren PE, Espinoza F, Svensson L (2008). Pediatric norovirus diarrhea in Nicaragua. J Clin Microbiol.

[44] Lekana-Douki SE, Kombila-Koumavor C, Nkoghe D, Drosten C, Drexler JF, Leroy EM (2015). Molecular epidemiology of enteric viruses and genotyping of rotavirus A, adenovirus and astrovirus among children under 5 years old in Gabon. Int J Infect Dis.

[45] Anders KL, Thompson CN, Thuy NTV, Nguyet NM, Tu LTP, Dung TTN, Phat VV, Van NTH, Hieu NT, Tham NTH (2015). The epidemiology and aetiology of diarrhoeal disease in infancy in southern Vietnam: A birth cohort study. Int J Infect Dis.

[46] Platts-Mills JA, Babji S, Bodhidatta L, Gratz J, Haque R, Havt A, McCormick BJJ, McGrath M, Olortegui MP, Samie A (2015). Pathogen-specific burdens of community diarrhoea in developing countries: A multisite birth cohort study (MAL-ED). Lancet Glob Health.

[47] Wheeler JG, Sethi D, Cowden JM, Wall PG, Rodrigues LC, Tompkins DS, Hudson MJ, Roderick PJ (1999). Study of infectious intestinal disease in England: Rates in the community, presenting to general practice, and reported to national surveillance. Br Med J.

[48] Sinclair MI, Hellard ME, Wolfe R, Mitakakis TZ, Leder K, Fairley CK (2005). Pathogens causing community gastroenteritis in Australia. Journal of Gastroenterology and Hepatology (Australia).

[49] Huhulescu S, Kiss R, Brettlecker M, Cerny RJ, Hess C, Wewalka G, Allerberger F (2009). Etiology of acute gastroenteritis in three sentinel general practices, Austria 2007. Infection.

[50] Kirk MD, Moffatt CRM, Hall GV, Becker N, Booy R, Heron L, MacIntyre R, Dwyer DE, Lindley R (2010). The burden of infectious gastroenteritis in elderly residents and staff of long-term care facilities, Australia. Infect Control Hosp Epidemiol.

[51] Ballard SB, Reaves EJ, Luna CG, Silva ME, Rocha C, Heitzinger K, Saito M, Apaza S, Espetia S, Blazes DL, et al., Epidemiology and genetic characterization of noroviruses among adults in an endemic setting, Peruvian Amazon Basin**,** 2004–2011. PLoS One. 2015, 10:7. 10.1371/journal.pone.0131646 PMC4498765 26161556

[52] Gauci C, Gilles H, Mamo J, Ruggieri FM, di Bartolo I, Barbara C, Cuschieri L (2010). The aetiology of infectious intestinal disease in the community in Malta. Malta Med J.

[53] Baumann-Popczyk A, Sadkowska-Todys M, Rogalska J, Stefanoff P (2012). Incidence of self-reported acute gastrointestinal infections in the community in Poland: A population-based study. Epidemiol Infect.

[54] Ahmed S, Ricketts P, Bergeron M, Jones W, Indar L (2013). Distribution, burden, and impact of acute gastroenteritis in Dominica, 2009–2010. J Health Popul Nutr.

[55] Ingram M, St. John J, Applewhaite T, Gaskin P, Springer K, Indar L (2013). Population-based estimates of acute gastrointestinal and foodborne illness in Barbados: A retrospective cross-sectional Study. J Health Popul Nutr.

[56] Verhoef L, Koopmans M, Van Pelt W, Duizer E, Haagsma J, Werber D, Van Asten L, Havelaar A (2013). The estimated disease burden of norovirus in the Netherlands. Epidemiol Infect.

[57] Moyo SJ, Gro N, Matee MI, Kitundu J, Myrmel H, Mylvaganam H, Maselle SY, Langeland N. Age specific aetiological agents of diarrhoea in hospitalized children aged less than five years in Dar es Salaam, Tanzania. BMC Pediatr. 2011;11 10.1186/1471-2431-11-19 PMC3050719 21345186

[58] Xue Y, Pan H, Hu J, Wu H, Li J, Xiao W, Zhang X, Yuan Z, Wu F (2015). Epidemiology of norovirus infections among diarrhea outpatients in a diarrhea surveillance system in Shanghai, China: a cross-sectional study. BMC Infect Dis.

[59] Ammon A, Makela P (2010). Integrated data collection on zoonoses in the European Union, from animals to humans, and the analyses of the data. Int J Food Microbiol.

[60] Agócs MM, Serhan F, Yen C, Mwenda JM, de Oliveira LH, Teleb N, Wasley A, Wijesinghe PR, Fox K, Tate JE (2014). WHO global rotavirus surveillance network: a strategic review of the first 5 years, 2008–2012. MMWR Morb Mortal Wkly Rep.

[61] Marshall JA, Bruggink LD (2006). Laboratory diagnosis of norovirus. Clin Lab.

[62] Yu J, Ye C, Lai S, Zhu W, Zhang Z, Geng Q, Xue C, Yang W, Wu S, Hall AJ (2017). Incidence of Norovirus-Associated Diarrhea, Shanghai, China, 2012–2013. Emerg Infect Dis.

